# Predicting Canine and Premolar Mesiodistal Crown Diameters Using Regression Equations

**DOI:** 10.1155/2021/9990417

**Published:** 2021-07-23

**Authors:** Jinan Eliewy Saloom, Arkan Muslim Al Azzawi, Mohammed Nahidh, Sajid Chaffat Auliawi Al-Mayahi, Baraa Sahib Mahdi

**Affiliations:** ^1^Department of Orthodontics, College of Dentistry, University of Baghdad, Baghdad, Iraq; ^2^Department of POP, College of Dentistry, University of Babylon, Babylon, Iraq; ^3^Ministry of Health, Baghdad, Iraq; ^4^Private Clinic, Karbala'a, Iraq

## Abstract

**Objectives:**

The current study aimed to predict the combined mesiodistal crown widths of maxillary and mandibular canines and premolars from the combined mesiodistal crown widths of maxillary and mandibular incisors and first molars.

**Materials and Methods:**

This retrospective study utilized 120 dental models from Iraqi Arab young adult subjects with normal dental relationships. The mesiodistal crown widths of all teeth (except the second molars) were measured at the level of contact points using digital electronic calipers. The relation between the sum mesiodistal crown widths of the maxillary and mandibular incisors and first molars and the combined mesiodistal crown widths of the maxillary and mandibular canines and premolars was assessed using Pearson's correlation coefficient test. Based on this relation, regression equations were developed to predict the sum widths of maxillary and mandibular canines and premolars; then, the predicted mesiodistal crown sum widths were compared with the actual one using a paired sample *t*-test.

**Results:**

Statistically, the predicted mesiodistal crown sum widths were nonsignificantly different from the actual ones.

**Conclusions:**

The combined mesiodistal widths of maxillary and mandibular canines and premolars can be predicted successfully from the combined mesiodistal widths of the maxillary and mandibular incisors and first molars with a high degree of accuracy reaching to more than 86%.

## 1. Introduction

During orthodontic assessment for space analysis in the period of mixed dentition, it is imperative to reach a precise prediction for the available space that will house the unerupted canines and premolars in order to develop an appropriate treatment plan for extraction or nonextraction therapy [1].

Precision, safety, and ease are crucial principles for a predictive method to become an element of the inclusive case analysis in current orthodontic practice [2].

Average teeth width was first studied by Dr. G. V. Black [3]. Due to individual variations in teeth size in different genders and race groups, Black's work was not particularly precise. Tanaka and Johnston [4] and Moyers [5] developed the two most popular methods to predict unerupted canine and premolar crown diameters.

However, these methods may not apply to all racial groups because they can give overestimated results. Many other methods utilizing regression analysis of maxillary or mandibular anterior teeth and first molar widths reported acceptable results in different racial groups [6–14].

In Iraq, several studies have been performed to predict canine and premolar widths using different methods [15–18]. The present study aimed to utilize combined mesiodistal crown widths of the maxillary and mandibular incisors and first molars to predict the combined mesiodistal crown widths of the maxillary and mandibular canines and premolars using simple regression analysis.

## 2. Materials and Methods

Approval for conducting this retrospective study was obtained from the scientific and ethical committees in the College of Dentistry, University of Baghdad, Iraq (Ref. no. 5, 1 Jan 2020).

The samples comprised 120 dental models from 120 Iraqi Arab subjects (60 males and 60 females). Those were selected from the archives of the Department of Orthodontics, College of Dentistry, University of Baghdad, according to specific criteria.

The eligibility criteria included the following: all subjects were aged 17–25 years with a full set of permanent teeth and normal skeletal and dental relations in three planes of space as indicated by the two figures method, assessing the facial heights and asymmetry and examining the molar, canine, and incisor relationships [19]. The teeth were sound with no massive caries, attrition, proximal restorations, or any other defects. Moreover, any model with defect of fractured tooth was excluded.

The teeth in the maxillary and mandibular arches from the first molar to the first molar on the other side were measured at the largest mesiodistal dimension using electronic digital calipers (Mitutoyo, Japan) with a sensitivity of 0.01 mm held perpendicular to the long axis of the teeth [20].

The major idea behind this study was to develop regression equations that were used to predict the sum mesiodistal crown diameters of canines and both premolars in both genders and arches based on the sum crown diameters of the maxillary and mandibular incisors and first molars in the mixed dentition stage in order to prevent further development of malocclusion like crowding or space loss of permanent teeth. This procedure will pass in four steps:

1. Assessing the relationships between the sum mesiodistal crown diameters of canines and both premolars with the sum crown diameters of the maxillary and mandibular incisors and first molars in both genders and jaws.
2. Verifying the gender differences for these variables.
3. Developing regression equations for both genders and arches that predict the sum mesiodistal crown diameters of canines and premolars from the sum crown diameters of the maxillary and mandibular incisors and first molars.
4. Comparing the predicted widths, gained from applying the regression equations, with the actual widths.

Data were analyzed using the SPSS program (version 25, IBM Co., USA). These analyses included the following:

(1) Descriptive statistics (mean values, standard deviations, and numbers/percentages)
(2) Inferential statistics that comprised the following:
  - Intraclass correlation coefficient (ICC) test to confirm intra- and interobserver reliabilities
  - Pearson's correlation coefficient test to determine the relation between sum mesiodistal widths of the maxillary and mandibular incisors and first molars with the sum mesiodistal widths of the maxillary and mandibular canines and premolars
  - Independent sample *t*-test to analyze gender differences
  - Simple regression analysis to establish regression equations
  - Paired sample *t*-test to test the difference between predicted and actual teeth widths in both genders

A probability value (*P* value) of more than 0.05 was considered to be a statistically nonsignificant difference.

## 3. Results

Firstly, interexaminer reliability was tested by measuring the mesiodistal crown diameters of all teeth anterior to the second molars in both arches on ten randomly selected models by two researchers (M. N. and B. S. M.), and the results of the intraclass correlation coefficient test indicated excellent reliability (0.93). On the other hand, the same measurements were repeated on the same ten models by the main examiner (B. S. M.) after two weeks in order to test the intraexaminer reliability which again indicated excellent reliability (0.96).

The relations between the combined width of the maxillary and mandibular incisors and 1^st^ molars with the combined width of the maxillary and mandibular canines and premolars are provided in Table 1. There were direct, strong, and highly significant relations between the measurements in both genders and arches (*P* ≤ 0.001).

Table 2 provides the mean values and standard deviations of measurements used in this study. In general, males had statistically higher significant mean values than females for all measurements (*P* ≤ 0.001).

The third step involved applying the simple regression analysis test, with combined width of the maxillary and mandibular incisors and 1^st^ molars representing the independent variable and the maxillary and mandibular canines and premolars combined crown diameters acting as dependent variables. The regression equations were obtained as presented in Table 3.

Based on the values gained from these equations, the predicted mesiodistal crown dimensions of the maxillary and mandibular canines and premolars were calculated and compared with the actual ones using a paired samples *t*-test (Table 4). The results revealed nonsignificant differences (*P* > 0.05).

Each predicted measurement was subtracted from the actual one in order to test the frequencies and percentages of cases lying within the limit of a 2 mm difference of over- and underestimating canine and premolar actual widths. More than 86% of cases were found to lie within this limit (Table 5).

## 4. Discussion

Predicting the combined widths of the maxillary and mandibular canines and premolars in the early mixed dentition stage is vital for preventing dental arch crowding. Methods for predicting unerupted teeth widths developed by Tanaka and Johnston [4] and Moyers [5] cannot be applied to all ethnic groups due to racial variations in teeth sizes. Many authors have used simple regression analysis to develop regression equations and, thus, predict the width of unerupted teeth from erupted teeth. This method is easy, highly accurate, uncomplicated, and does not require *X*-rays or probability tables.

In the current study, the maxillary and mandibular incisors and first molars were chosen to predict the widths of the maxillary and mandibular canines and premolars because they erupted early in the oral cavity.

The relations between the combined mesiodistal widths of the maxillary and mandibular incisors and first molars with combined mesiodistal crown dimensions of the maxillary and mandibular canines and premolars are presented in Table 1. Generally, there was a direct, strong, and highly significant correlation between these parameters in both genders and arches and comes in agreement to that reported in other studies [4, 17, 18].

To verify gender differences for the measured variables, an independent sample *t*-test was applied. Significantly higher mean values were found for male teeth (Table 2), confirming the fact that males have larger teeth than females [2, 5, 17, 18]; thus, a specific regression equation was developed for each gender. This equation was applied as *Y* = *a* + *b X*, where “*Y*” is the sum mesiodistal crown widths of the mandibular or maxillary permanent canines and premolars, “*X*” is the sum mesiodistal crown widths of the maxillary and mandibular incisors and first molars, “*a*” is a constant, and “*b*” is the regression coefficient (Table 3).

After computing the predicted widths from the developed regression equations, a paired sample *t*-test was applied to compare actual and predicted measurements. A nonsignificant difference was shown between the predicted and actual mesiodistal crown dimensions of both the maxillary and mandibular canines and premolars (Table 4); this result agrees with previous studies [6–9, 11–13, 17, 18]. The mean differences between the measurements were clinically and statistically insignificant.

The actual measurements were subtracted from the predicted ones in order to determine the new method's precision. More than 86% of predicted cases were found to lie within a limit of 2 mm in either direction. Many authors have suggested that an overestimation of only 1 mm beyond the actual widths of the permanent canine and premolars on each side of the arch might not substantially alter extraction or nonextraction judgments [8–10]. A small number of cases either over-or underestimated the actual measurements have been recorded. Hence, the method used in the current study appears to be more reliable for application.

Overestimation is considered to be superior to underestimation in order to prevent a lack of space; however, this approach could lead to unnecessary teeth extractions for some patients. Decisions regarding extraction or nonextraction therapy would not be critically influenced by a one mm overestimation per side of the arch beyond the permanent canine and premolar actual widths [5, 9, 10].

The major limitation of the present study was the relatively small sample size as normal occlusal and skeletal relationships are hard to find. On the other hand, the main drawback of the prediction method is that it is not completely accurate, and the actual size of unerupted teeth might be over- or underestimated, so larger sample size is required.

## 5. Conclusions

From a clinical and statistical point of view, the predicted mesiodistal crown widths showed a nonsignificant difference from the actual ones. Accordingly, the combined mesiodistal widths of the maxillary and mandibular canines and premolars can be predicted with high accuracy, reaching up to 86% from the sum mesiodistal widths of the maxillary and mandibular incisors and first molars with no need for X-rays or probability tables.

## Figures and Tables

**Table 1:** Relation between combined mesiodistal crown diameters of the maxillary and mandibular incisors and first molars with combined mesiodistal crown diameters of the maxillary and mandibular canines and premolars.

MDMMIFM		MDCP			
		Maxillary		Mandibular	
		Males	Females	Males	Females
	*r*	0.764	0.712	0.755	0.709
	*P*	≤0.001	≤0.001	≤0.001	≤0.001

MDMMIFM: mesiodistal diameter of the maxillary and mandibular incisors and first molars. MDCP: mesiodistal diameter of the canine and premolars.

**Table 2:** Descriptive statistics and gender differences for measured variables.

Measurements (mm)	Gender	Descriptive statistics		Gender difference	
		Mean	S.D.	*t*-test	*P* value
MDMMIFM	Males	97.386	4.353	4.841	≤0.001
	Females	93.783	3.586		

Maxillary MDCP	Males	43.513	1.875	4.368	≤0.001
	Females	41.946	1.972		

Mandibular MDCP	Males	42.502	1.869	4.406	≤0.001
	Females	40.965	1.872		

**Table 3:** Regression equations.

Genders	For maxillary arch	For mandibular arch
Males	MDCP = 11.457 + 0.329 × MDMMIFM	MDCP = 10.940 + 0.324 × MDMMIFM
Females	MDCP = 5.204 + 0.392 × MDMMIFM	MDCP = 6.548 + 0.367 × MDMMIFM

**Table 4:** Descriptive statistics and comparison between predicted and actual combined mesiodistal crown dimensions of the maxillary and mandibular canines and premolars.

Gender	Arch	Descriptive statistics				Difference between predicted and actual MDCP		
		Actual MDCP		Predicted MDCP				
		Mean	S.D.	Mean	S.D.	Mean difference	*t*-test	*P* value
Males	Maxillary	43.513	1.875	43.497	1.432	0.016	0.099	0.922
	Mandibular	42.502	1.869	42.493	1.410	0.009	0.054	0.957

Females	Maxillary	41.946	1.972	41.967	1.406	−0.021	−0.116	0.908
	Mandibular	40.965	1.872	40.966	1.316	−0.001	−0.008	0.993

**Table 5:** Frequencies and percentages of cases that lied within the 2 mm limit of over- and underestimating the actual combined width of the canines and premolars.

Arch	Gender	Within limit	Overestimated	Underestimated
Maxillary	Males	52 (86.7%)	4 (6.7%)	4 (6.7%)
	Females	53 (88.3%)	3 (5%)	4 (6.7%)

Mandibular	Males	56 (93.3%)	2 (3.3%)	2 (3.3%)
	Females	52 (86.7%)	4 (6.7%)	4 (6.67%)

## Data Availability

The data that support the findings of this study are available from the corresponding author upon reasonable request.
